# The triumvirate of signaling molecules controlling *Toxoplasma* microneme exocytosis: Cyclic GMP, calcium, and phosphatidic acid

**DOI:** 10.1371/journal.ppat.1007670

**Published:** 2019-05-23

**Authors:** Hayley E. Bullen, Hugo Bisio, Dominique Soldati-Favre

**Affiliations:** 1 Burnet Institute, Melbourne, Victoria, Australia; 2 Department of Microbiology and Molecular Medicine, CMU, University of Geneva, Geneva, Switzerland; University of Wisconsin Medical School, UNITED STATES

## Abstract

To elicit effective invasion and egress from infected cells, obligate intracellular parasites of the phylum Apicomplexa rely on the timely and spatially controlled exocytosis of specialized secretory organelles termed the micronemes. The effector molecules and signaling events underpinning this process are intricate; however, recent advances within the field of *Toxoplasma gondii* research have facilitated a broader understanding as well as a more integrated view of this complex cascade of events and have unraveled the importance of phosphatidic acid (PA) as a lipid mediator at multiple steps in this process.

## The signaling node

**Fig 1:**
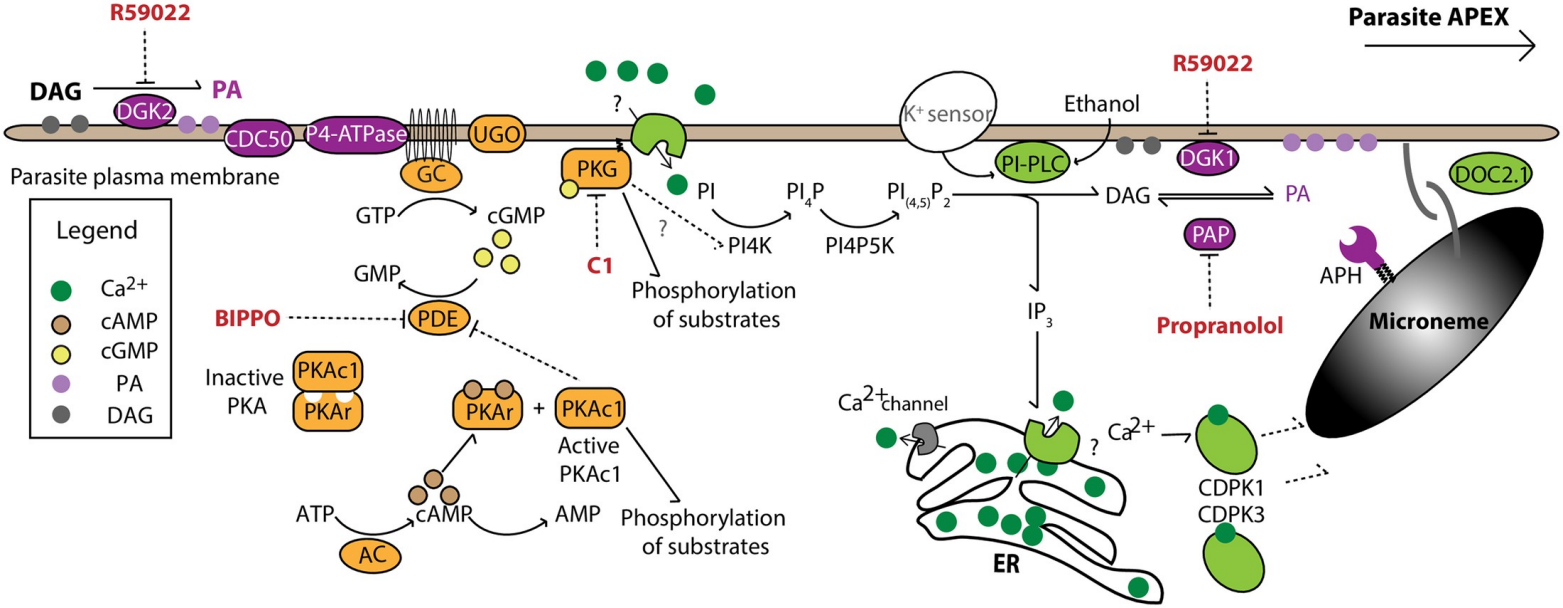
Schematic of the signaling cascade underpinning cGMP, calcium, and PA generation at the parasite pellicle. Activation of GC at the parasite plasma membrane in response to DGK2 activation and subsequent PA production promotes the formation of cGMP. cGMP serves to activate PKG, which in turn promotes the formation of PI-PLC substrates. cGMP production is regulated by PDE, which is regulated by the activity of the PKAc1. PKAc1 is itself regulated by PKA regulatory domain, which binds AC-generated cAMP. PI-PLC converts PI(_4,5_)P2 to IP3 and DAG. IP3 is thought to stimulate the release of calcium, likely from ER stores, whereas DAG is converted by DGK1 into PA. PA bound by APH facilitates DOC2.1-mediated fusing of the micronemes to the parasite surface and thus their exocytosis. AC, adenylate cyclase; APH, acylated pleckstrin homology domain–containing protein; BIPPO, 5-benzyl-3-isopropyl-1H-pyrazolo[4,3-d]pyrimidin-7(6H)-one; C1, compound 1; cAMP, cyclic adenosine monophosphate; CDC50.1, cell division control protein 50.1; CDPK, calcium-dependent protein kinase; cGMP, cyclic guanosine monophosphate; DAG, diacylglycerol; DGK, DAG kinase; ER, endoplasmic reticulum; DOC2.1, double C2 domain–containing protein 1; GC, guanylate cyclase; GTP, guanosine triphosphate; IP3, inositol triphosphate; PA, phosphatidic acid; PAP, PA phosphatase; PDE, phosphodiesterase; PI, phosphoinositol; PI(_4,5_)P2, phosphatidylinositol 4,5-bisphosphate; PI4K, phosphatidylinositol 4-kinase; PI_4_P, phosphatidylinositol 4-phosphate; PI4P5K, phosphatidylinositol 4-phosphate 5-kinase; PI-PLC, phosphoinositide-phospholipase C; PKAc1, protein kinase A catalytic 1 domain; PKAr, PKA regulatory subunit; PKG, protein kinase G; UGO, unique GC organizer.

At any point during intracellular replication, deleterious environmental changes resulting in a loss of host-cell integrity can trigger *T*. *gondii* tachyzoite egress from infected cells via activation of microneme exocytosis and the actomyosin system. Extrinsic and intrinsic signals are likely to govern parasite egress from infected cells; however, the studies performed to date have implicated only specific extrinsic stimuli including low potassium (K^+^), low pH [1, 2], and serum albumin [3]. During the intracellular cycle, *T*. *gondii* tachyzoites are surrounded by a parasitophorous vacuole membrane (PVM), which is permeable to small molecules, and changes in K^+^ or H^+^ levels are sensed by the parasite through unknown mechanisms to promote microneme secretion. Interestingly, exposure to an acidic environment can overcome a high potassium–induced block in microneme exocytosis, implying that K^+^ and pH are sensed by different receptors or that pH sensing is downstream of K^+^ detection [2]. These extrinsic signals feed into a pathway within which phosphoinositide-phospholipase C (PI-PLC) forms a signaling node, hydrolyzing phosphatidylinositol 4,5-bisphosphate (PI_[4,5]_P_2_) to generate diacylglycerol (DAG) and inositol triphosphate (IP_3_) to produce PA and to mobilize calcium, respectively, to ultimately trigger microneme exocytosis [4]. Further feeding this pathway are diverse signaling events carried out by cyclic nucleotides (Fig 1 and Table 1).

**Table 1:** Key mediators of cyclic nucleotides, calcium, and PA regulation in *T*. *gondii*.

**PA**	**Inactivation**	**Phenotype**	**References**
DGK1	R59022/iKD (DD system)	Reduced microneme secretion, egress defect	[4]
	iKD (Tet system)/KO	Loss of plasma membrane integrity	[4]
APH	iKD (Tet system)	Defect in microneme secretion	[4, 24]
	iKO (Cre recombinase)	Egress defect	[4]
GAC	iKD (Tet system)	Defect in motility, invasion, egress	[28]
DGK2	iKD (Tet system)/KO	Delay in natural egress	[26]
P4-ATPase (GC)	Catalytic inactivation (site-specific mutagenesis)	ATPase activity is essential for survival	[27]
	P2A skip peptide	Mislocalization of GC catalytic domains	[2]
CDC50.1	iKD (AID system)	Delay in natural egress/BIPPO-induced egress	[26]
**Cyclic nucleotides**	**Inactivation**	**Phenotype**	**References**
ACalpha1–3	KO	Mild to no fitness cost	[27]
ACbeta	KO	Strong fitness cost	[27]
	iKD (Tet system)	No fitness cost	[9]
ACs	ACbeta iKD (Tet system) + ACalpha KO	Host-cell destruction/invasion defect	[9]
GC	iKD (AID system)	Defect in microneme secretion/failure to disconnect	[26, 27]
UGO	iKD (AID system)	GC mislocalization/defect in microneme secretion	[26]
PKG	iKD (AID system)/Inh1/Inh2	Defect in microneme secretion	[5, 15, 18]
	C1/C2	Defect in microneme secretion/actomyosin motor activation	[23]
PKAr	iKD (DD system)	Intracellular growth defect	[9]
	Conditional overexpression of cAMP-binding domain point mutant (DD system)	Premature egress/restless invasion/increased microneme secretion in intracellular buffer	[9]
PKAc1	iKD (Tet system)	Premature egress/restless invasion	[9, 10]
	Conditional overexpression (DD system)	Intracellular growth defect	[9]
	H89 or KT5270	Premature egress	[9]
PDEs	BIPPO/zaprinast	Defect in microneme secretion	[8]
**Calcium**	**Inactivation**	**Phenotype**	**References**
CDPK1	iKD (Tet system)/3-MB-PP1	Defect in microneme secretion/actomyosin motor activation	[6, 23]
CDPK3	KO/3-MB-PP1	Egress upon calcium ionophore treatment	[6, 18, 19]
DOC.2	Cold-sensitive mutant	Defect in microneme secretion	[25]
PI-PLC	U73122	Defect in microneme secretion	[4]
	iKD (Tet system)	Loss of plasma membrane integrity	[4]

Abbreviations: 3-MB-PP1, 4-amino-1-tert-butyl-3-(3-methylbenzyl)pyrazolo[3,4-d]pyrimidine; AC, adenylate cyclase; APH, acylated pleckstrin homology domain–containing protein; AID, auxin inducible degron; BIPPO, 5-benzyl-3-isopropyl-1H-pyrazolo[4,3-d]pyrimidin-7(6H)-one; C1, compound 1; C2, compound 2; cAMP, cyclic adenosine monophosphate; CDC50.1, cell division control protein 50.1; CDPK, calcium-dependent protein kinase; DD, destabilization domain; DGK, diacylglycerol kinase; DOC.2, double C2-domain–containing protein 1; GAC, glideosome-associated connector; GC, guanylate cyclase; iKD, inducible knock-down; Inh1, inhibitor 1; Inh2, inhibitor 2; KO, knock-out; IP_3_, inositol triphosphate; P2A, 2A self-cleaving peptide; PA, phosphatidic acid; PDE, phosphodiesterase; PI-PLC, phosphoinositide-phospholipase C; PKAc1, protein kinase A catalytic 1 domain; PKAr, PKA regulatory subunit; PKG, protein kinase G; Tet, Tet-repressor inducible knock-down; UGO, unique GC organizer.

## Cross talk between cyclic nucleotides

Cyclic nucleotides cyclic adenosine monophosphate (cAMP) and cyclic guanosine monophosphate (cGMP) are important second messengers with diverse roles in eukaryotic cells. Within the Apicomplexa, cGMP is a vital upstream mediator of signaling events leading presumably to PI-PLC activation and subsequent microneme secretion. Protein kinase G (PKG) senses fluctuations in cGMP levels, and its association with the parasite plasma membrane by acylation is a necessary modification to function in controlling motility and invasion [5]. PKG critically participates in the mobilization of intracellular calcium, and its specific inhibition with either compound 1 (tri-substituted pyrrole 4-[2-(4-fluorophenyl)-5-(1-methylpiperidine-4-yl)-1H-pyrrol-3-yl]pyridine) or, less selectively, compound 2 (4-[7-[(dimethylamino) methyl]-2-(4-fluorophenyl) imidazo[1,2-a] pyridin-3-yl] pyrimidin-2-amine) has been shown to impact microneme secretion and tachyzoite egress [6, 7].

Cyclic nucleotides are degraded by phosphodiesterases (PDEs), of which there are 18 putative genes in *T*. *gondii* [8]. Importantly, inhibition of apicomplexan PDEs with either the human PDE5 inhibitor zaprinast [4, 6] or the potent PDE inhibitor 5-benzyl-3-isopropyl-1H-pyrazolo[4,3-d]pyrimidin-7(6H)-one (BIPPO) [8] has been shown to induce microneme secretion and parasite egress [4, 6]. BIPPO impacts both cAMP- and cGMP-dependent processes, suggesting that it may inhibit both cAMP- and cGMP-specific PDE isoform(s) [8].

Also involved in egress and invasion is *T*. *gondii* cAMP-dependent protein kinase A catalytic subunit 1 (PKAc1), which is targeted to the parasite pellicle via its association with the dually acylated PKA regulatory subunit (PKAr) [9]. Remarkably, PKAc1 inactivation results in acidification-dependent premature egress followed by successive invasion events leading to host-cell destruction [9]. The host-cell destruction correlates with the inability of PKAc1-depleted parasites to suppress Ca^2+^ signaling upon host-cell invasion [10] and switch from the motile to the replicative stage. Compound 1 blocks premature egress induced by either PKAc1 inactivation or environmental acidification, suggesting that pH and PKAc1 balance the level of cGMP to control egress. Concordantly, changes in the phosphorylation profile of a cGMP-PDE following PKAc1 inactivation might play a role in the interplay between cAMP and cGMP signaling, leading to cross talk between PKA and PKG pathways [9] (Table 1).

## Calcium sensing and calcium-dependent protein kinase responses

Downstream of PKG, PKA, and PI-PLC activity is IP_3_ production and the ensuing release of calcium (Ca^*2+*^), a process previously reported to be sensitive to IP_3_ receptor inhibitors [11]. Despite the strong pharmacological evidence for their existence, canonical genes encoding IP_3_ receptors cannot be identified. Apicomplexan parasites possess several putative Ca^2+^ stores, including the acidocalcisomes, the mitochondrion, the inner membrane complex (IMC), and most relevantly, the endoplasmic reticulum (ER). Treatment of parasites with the sarcoplasmic/ER Ca^2+^-ATPase (SERCA) inhibitor thapsigargin has been shown to prevent reentry of Ca^2+^ in the ER and to stimulate microneme exocytosis, presumably by blocking the influx of leaked Ca^2+^ from the ER [12]. The recent advance in developing genetically encoded Ca^2+^ indicators has offered a powerful tool to visualize Ca^2+^ during egress and was exploited to identify compounds modulating Ca^2+^ signaling via a cell-based phenotypic screen for compounds that modulate Ca^2+^ signaling [13–15]. Additionally, Ca^2+^ is proposed to be mobilized in response to abscisic acid (ABA) via the production of cyclic ADP (cADP) ribose [16]. Although *T*. *gondii* possesses ADP ribosyl cyclase and hydrolase [17] and a putative ABA-binding G-protein–coupled receptor (GPCR) receptor (G-protein–coupled receptor 89 [GPR89], TGGT1_286490), no pathway for the biosynthesis of ABA or ryanodine receptor could be found.

The Apicomplexa lack typical Ca^2+^ effector kinases (protein kinase C [PKC] and Ca^2+^/calmodulin-dependent protein kinase [CaMK]) and instead utilize phylum-specific calcium-dependent protein kinases (CDPKs) that are activated by the direct binding of Ca^2+^ to their EF-hands. Among the 14 genes coding for *T*. *gondii* CDPKs, CDPK1 was shown to be critical for microneme secretion. CDPK3, the only other member implicated to date in egress is dispensable and plays a more specific role in parasite egress [6, 18, 19]. Potential CDPK1 and CDPK3 substrates have been identified; however, the precise targets of the CDPKs and their specific contribution to microneme secretion are yet to be fully described [20–22]. Of relevance, CDPK1 plays a role not only in microneme secretion but also in the activation of the actomyosin system as well as the extrusion of the conoid, an apical motile organelle composed of tubulins fibers and presumed to be important for microneme exocytosis [23] (Fig 1 and Table 1).

## PA sensing in microneme exocytosis and natural egress

The production of IP_3_ by PI-PLC and concurrent Ca^2+^ mobilization preceding microneme secretion is also linked to the generation of DAG, which can be interconverted to PA through the action of DAG kinases (DGKs), and PA phosphatases (PAPs) [4]. In mammalian cells, PA has been linked to exocytosis, and similarly, blocking PA production with either specific DGK inhibitors or through conditional depletion of the plasma membrane–associated DGK1 reduces PA production and blocks microneme secretion in *T*. *gondii* [4]. Moreover, microneme secretion can be induced by treatment of *T*. *gondii* with the PAP inhibitor propranolol, implying that the ensuing buildup of PA facilitates exocytosis [4]. Although the signaling pathway culminating in microneme release is slowly being unraveled, little is known about the actual fusion event at the parasite plasma membrane. Work delineating the importance of PA signaling at the parasite plasma membrane has, however, revealed the presence of a novel, PA-binding acylated pleckstrin homology domain–containing protein (APH) at the surface of the micronemes [4]. Conditional depletion of the *APH* gene resulted in a selective block in microneme secretion, leading to impairments in parasite motility, invasion, and egress [4]. APH is able to cluster multiple phosphate head-groups at the bilayer-binding surface [24], which is presumably critical to bringing membranes together during microneme exocytosis to induce SNAP receptor (SNARE)-mediated fusion [4]. SNARES and C2 domain–containing proteins mediate vesicle–membrane fusion in diverse cell types in response to Ca^2+^ and membrane curvature. In both *T*. *gondii* and *Plasmodium falciparum*, a conserved C2 domain–containing protein termed DOC2.1 has been shown to play a role in microneme secretion, as well as the associated events of egress, motility, and invasion [25]. Although DOC2.1 is yet to be localized, it has been proposed to mediate microneme release by assisting SNARE-like protein/complex formation in a Ca^2+^-dependent manner to prompt microneme–plasma membrane fusion [25] (Fig 1 and Table 1).

In addition to DGK1, *T*. *gondii* expresses DGK2, which is secreted into the parasitophorous vacuole (PV) and is also implicated in PA signaling events leading to egress [26]. PA produced in the PV serves as an intrinsic signal to elicit natural egress, which occurs in a coordinated manner after approximately 5 to 6 cycles of endodyogeny. Parasites lacking DGK2 presumably fail to accumulate intravacuolar PA and exhibit a natural egress defect resulting in the formation of enlarged vacuoles that eventually rupture mechanically. PA acts upstream of (or directly activates) a large guanylate cyclase (GC) receptor, which is uniquely conserved in alveolates and ciliates. The GC comprise one P4-ATPase and two GC catalytic domains and crucially initiates cGMP-mediated signaling in *T*. *gondii* [27]. Remarkably, the assembly of this atypical GC at the parasite plasma membrane critically depends on two protein cofactors, the cell division control protein 50.1 (CDC50.1), known to act as a chaperone/regulatory subunit for the P4-ATPase, and a unique GC organizer (UGO), which is necessary for the traffic and activity of GC, respectively. The current model suggests that this complex serves as a versatile signaling platform to integrate both intrinsic PA lipid signaling and other extrinsic signals [26] (Fig 1).

## PA and the glideosome-associated connector

Motility and host-cell entry and egress involve a parasite-derived molecular motor termed the glideosome, components of which are located between the parasite plasma membrane and IMC, as well as specific parasite surface adhesins. An armadillo repeat–containing protein identified as glideosome-associated connector (GAC) was found to be crucial for motility, invasion, and egress without impacting microneme secretion [28]. GAC not only acts as a connector between F-actin and the tail of the transmembrane microneme protein 2 (MIC2) adhesin but also possesses a PA-binding pleckstrin homology domain [28], which is likely involved in binding GAC to the parasite apex during motility following the DGK1-related up-regulation of PA. The dual binding of GAC to the MIC2 tail and PA presumably ensures higher affinity and selectivity for the secreted adhesin by combining two weak binding interactions, engendering strong binding. This finding not only presents a crucial link between the parasite surface adhesins and the underlying actomyosin network but also adds to the importance of PA signaling during invasion-related events not solely limited to microneme exocytosis.

## Conclusion

Microneme exocytosis is the culmination of a complex series of events that are slowly being unraveled. Further investigations into the roles of CDPKs, PI-PLC regulation, cyclic nucleotides, and integration of potassium, pH, and PA sensing will be required to give a global overview of how tachyzoites govern natural and induced egress as well as invasion to perpetuate their pathogenicity.
